# Effects of Transcranial Direct Current Stimulation Versus Sham Stimulation on Brain-Derived Neurotrophic Factor and Clinical Outcomes: A Systematic Review and Meta-Analysis

**DOI:** 10.3390/jcm15186992

**Published:** 2026-09-09

**Authors:** Karam Addas, Ameen Aldabbagh, Jana Balloura, Ahmad Al Raiy, Rian M. Balafkhar, Lama S. Othman, Kadi Aldowairej, Osama Abdullah Al-Amoudi, Meshari M. Alotaibi, Fahad Mohammed Aldehaim, Hammam Sibyani, Bassam M. Alshahrani, Abdulrahman Alnwiji, Yasir Jasim, Ayman M. A. Mohamed

**Affiliations:** 1 College of Medicine, Alfaisal University, Riyadh 11533, Saudi Arabia; kaddas@alfaisal.edu (K.A.); aaldabbagh01@alfaisal.edu (A.A.); ahmadalraiy8@gmail.com (A.A.R.); hsibyani@alfaisal.edu (H.S.); aalnwiji@alfaisal.edu (A.A.); anabreesy@gmail.com (Y.J.); 2 College of Medicine, AlMaarefa University, Al Diriyah, Riyadh 13713, Saudi Arabia242220842@student.um.edu.sa (K.A.); 3 Faculty of Medicine, Bahçeşehir University, Istanbul 34734, Turkey; alamoudiosama10@gmail.com; 4 Faculty of Medicine, Shaqra University, Dawadmi 11911, Saudi Arabia; altiby10@gmail.com (M.M.A.); dartfhd@gmail.com (F.M.A.); 5 Faculty of Medicine, Shaqra University, Shaqra 15526, Saudi Arabia

**Keywords:** transcranial direct current stimulation, tDCS, brain-derived neurotrophic factor, BDNF, pain, pain catastrophizing, working memory, inflammatory biomarkers, neuroplasticity

## Abstract

**Purpose**: This study aimed to evaluate the effects of active transcranial direct current stimulation (tDCS) compared with sham stimulation on circulating brain-derived neurotrophic factor (BDNF), pain-related outcomes, working memory, and inflammatory and neuroplasticity biomarkers. **Methods**: PubMed/MEDLINE, Embase, the Cochrane Central Register of Controlled Trials, Web of Science, and ClinicalTrials.gov were searched from inception to May 2026. Randomized and controlled clinical trials comparing active with sham tDCS in adults were included. The primary outcome was post-treatment BDNF concentration. Secondary outcomes included pain intensity, pain catastrophizing, pain-related disability and functional interference, working memory, and circulating inflammatory or neuroplasticity biomarkers. Risk of bias was assessed using the revised Cochrane risk-of-bias tool. Mean differences (MDs) with 95% confidence intervals (CIs) were pooled using random-effects models. Serum and plasma BDNF were analyzed separately. **Results**: Twenty-one studies were included in the systematic review, of which 15 contributed to at least one quantitative synthesis and 12 study comparisons contributed to the primary BDNF analyses. Active tDCS did not significantly affect serum BDNF (MD = −0.01 [−0.15, 0.13], *p* = 0.890) or plasma BDNF (MD = 0.02 [−0.25, 0.29], *p* = 0.870) compared with sham stimulation. In contrast, active tDCS was associated with lower pain intensity (MD = −1.79 [−2.34, −1.23], *p* < 0.00001). No statistically significant difference was observed in the PCS total score or in the helplessness, magnification, or rumination subscales, although the point estimates generally favored active tDCS. For pain-related disability and functional interference, active tDCS was associated with lower PCP:S total scores and lower emotional interference, whereas no significant differences were observed for interference with daily activities or pain frequency. No significant differences were observed for working memory, interleukins, tumor necrosis factor-alpha, glial cell line-derived neurotrophic factor, interleukin-18, or soluble tumor necrosis factor receptors. Substantial heterogeneity was present in several pain-related and inflammatory biomarker analyses. **Conclusions**: Active tDCS was not associated with consistent differences in circulating serum or plasma BDNF or in the assessed peripheral inflammatory and neuroplasticity biomarkers compared with sham stimulation; however, these null peripheral findings should not be interpreted as evidence of absent central neuroplastic effects, because circulating measures may not adequately capture localized or transient biological changes within the central nervous system. Active tDCS was associated with improvements in some pain-related outcomes; however, the limited number of studies, substantial heterogeneity, and low certainty of the available evidence preclude firm conclusions regarding its clinical effectiveness for pain management.

## 1. Introduction

Transcranial direct current stimulation (tDCS) is a non-invasive neuromodulation technique that delivers a weak electrical current through electrodes positioned on the scalp. The applied current alters neuronal membrane polarization and modulates cortical excitability without directly inducing action potentials. Repeated stimulation may produce effects that persist beyond the treatment period through changes in synaptic plasticity, functional connectivity, neurotransmission, cerebral perfusion, glial activity, and immune-related signaling [1]. Its relatively simple application, portability, and favorable tolerability have supported its use across neurological, psychiatric, cognitive, and chronic pain conditions, including the development of remotely supervised and home-based protocols [2]. Nevertheless, the magnitude of the clinical response varies considerably and may be influenced by the stimulation target, electrode montage, current intensity, session duration, treatment frequency, clinical population, and concurrent interventions [3,4,5,6].

Brain-derived neurotrophic factor (BDNF) is an essential regulator of neuronal survival, differentiation, dendritic growth, synaptic transmission, learning, and memory [7]. Mature BDNF predominantly activates the tropomyosin receptor kinase B pathway, promoting neuronal survival and synaptic strengthening, whereas its precursor form, pro-BDNF, may exert different effects through the p75 neurotrophin receptor [7]. Because the prolonged effects of tDCS are thought to involve activity-dependent neuroplasticity, circulating BDNF has been proposed as a potential marker of its biological action and treatment response. Other inflammatory and neuroplasticity-related molecules, including interleukins, tumor necrosis factor-alpha, glial cell line-derived neurotrophic factor, and soluble tumor necrosis factor receptors, may also contribute to the physiological response to neuromodulation [8,9].

The biological rationale for investigating circulating BDNF after tDCS derives primarily from experimental evidence linking BDNF signaling to activity-dependent synaptic plasticity. Preclinical studies have shown that tDCS can modify BDNF expression and BDNF-dependent plasticity within stimulated brain regions [10,11,12]. Circulating BDNF has therefore been investigated in clinical studies as an accessible candidate biomarker of neuroplasticity-related responses to stimulation. Nevertheless, peripheral BDNF should not be considered a direct surrogate of cortical BDNF activity. Serum concentrations are strongly influenced by BDNF stored and released by platelets, while plasma measurements reflect a different circulating compartment, and the extent to which changes in either matrix correspond to BDNF activity within the central nervous system remains uncertain [13,14,15].

However, the interpretation of peripheral BDNF remains complex. Concentrations vary between serum and plasma and are influenced by platelet release, blood collection procedures, clotting duration, centrifugation, storage conditions, and assay characteristics [13,14,15,16]. Commercial assays may also differ in their ability to distinguish mature BDNF from pro-BDNF, resulting in substantial variation between reported concentrations [17,18]. In addition, sham-controlled clinical trials have reported increases, decreases, or no meaningful changes in BDNF following active tDCS [19,20,21,22,23,24].

Similar inconsistencies have been observed for inflammatory biomarkers and for clinical outcomes such as pain, pain catastrophizing, disability, cognition, and working memory [9,25]. Differences in the underlying condition, biological sample, assay method, stimulation protocol, co-intervention, treatment duration, and timing of outcome assessment may contribute to these conflicting findings [3,8].

Circulating BDNF has been investigated across diverse clinical applications of tDCS, including psychiatric, neurological, cognitive, and chronic pain populations [19,20,21,22,23,24]. Accordingly, the present review adopted a transdiagnostic approach rather than restricting eligibility to a single diagnosis. The objective was not to estimate disease-specific efficacy, but to determine whether active tDCS produces a reproducible peripheral BDNF response compared with sham stimulation across different clinical populations. The pooled estimates were therefore intended to represent average transdiagnostic effects, while differences in diagnosis, stimulation protocol, and biomarker methodology were considered potential sources of effect modification.

Although tDCS has been widely investigated, the available evidence regarding its effects on circulating BDNF and related biomarkers remains fragmented. Previous studies have generally included small samples, heterogeneous populations, and variable stimulation and laboratory procedures. It is therefore uncertain whether active tDCS produces a consistent change in serum or plasma BDNF compared with sham stimulation and whether these biological effects correspond with improvements in clinically relevant outcomes. The current systematic review and meta-analysis aimed to evaluate the effects of active tDCS compared with sham stimulation on circulating BDNF concentrations. Secondary objectives were to assess its effects on pain intensity, pain catastrophizing, pain-related disability and function, working memory, and inflammatory and neuroplasticity-related biomarkers.

## 2. Methods

This systematic review and meta-analysis was conducted in accordance with the Preferred Reporting Items for Systematic Reviews and Meta-Analyses recommendations and the methodological guidance of the Cochrane Handbook [26,27,28]. The PRISMA checklist can be found in Supplementary File S1. The review was prospectively registered with the International Prospective Register of Systematic Reviews (PROSPERO; CRD420261448614).

### 2.1. Search Strategy and Information Sources

Because the primary objective was to evaluate whether tDCS produces a generalizable peripheral BDNF response, eligibility was intentionally not restricted by clinical diagnosis; the analysis therefore represents a transdiagnostic comparison of active versus sham tDCS.

A comprehensive literature search was conducted in PubMed/MEDLINE, Embase, the Cochrane Central Register of Controlled Trials, and Web of Science from database inception to May 2026. ClinicalTrials.gov and the reference lists of the eligible studies and relevant reviews were also screened to identify additional or unpublished trials. The search strategy combined terms related to transcranial direct current stimulation, sham stimulation, BDNF, and the clinical and biological outcomes of interest. The principal search terms included: (“transcranial direct current stimulation” OR “tDCS”) AND (“sham stimulation” OR “sham tDCS” OR placebo) AND (“brain-derived neurotrophic factor” OR “BDNF” OR pain OR “pain catastrophizing” OR cognition OR “working memory” OR biomarker OR cytokine OR interleukin OR “tumor necrosis factor”). The search syntax was adapted according to the requirements of each database.

### 2.2. Study Selection

After removing duplicate records, two reviewers independently screened the titles and abstracts of the retrieved articles using Covidence. Potentially eligible reports were then evaluated through full-text screening. Any disagreement was resolved through discussion, and unresolved conflicts were referred to a third reviewer. No language restrictions were applied during the search or study-selection process. ClinicalTrials.gov records were screened to identify completed unpublished trials as well as ongoing studies. Completed trials were eligible for inclusion when sufficient outcome data were available either in the registry record or an associated publication. Ongoing trials and completed trials without available outcome data were not included in the quantitative synthesis because no analyzable treatment-effect data were available.

- Population: Adults aged 18 years or older, including healthy participants and patients with any clinical condition.
- Intervention: Active tDCS delivered using any electrode montage, current intensity, stimulation duration, number of sessions, or treatment setting. Studies involving a co-intervention were eligible when the same co-intervention was provided to the sham group.
- Comparator: Sham tDCS, either alone or accompanied by an identical background intervention. Comparisons involving an additional active drug were excluded unless the drug intervention was balanced by an equivalent placebo or the same treatment in the comparator group.
- Outcomes. The primary outcome was post-treatment BDNF concentration. Secondary outcomes included pain intensity, pain catastrophizing, pain-related disability and interference, working memory, adverse events, and inflammatory or neurobiological biomarkers, including IL-1, IL-6, IL-8, IL-10, IL-12p70, IL-18, TNF-α, GDNF, sTNFR1, and sTNFR2.
- Study design: Randomized or controlled clinical trials with parallel-group or crossover designs were eligible. Reviews, meta-analyses, case reports, conference abstracts without sufficient data, protocols, animal studies, and in vitro studies were excluded.

Multiple publications originating from the same registered trial were assessed for participant overlap. Reports were treated as separate studies only when the available information supported the use of independent participant samples. Otherwise, the most complete report was selected for the relevant outcome.

### 2.3. Quality Assessment & Certainty of Evidence

The risk of bias of the included randomized trials was independently evaluated by two reviewers using the revised Cochrane risk-of-bias tool for randomized trials (RoB 2) [28]. The assessment covered bias arising from the randomization process, deviations from the intended interventions, missing outcome data, measurement of the outcome, and selection of the reported result. Each study was classified as having a low risk of bias, some concerns, or a high risk of bias. For crossover studies, design-specific concerns, including treatment sequence, adequacy of the washout period, potential carryover and period effects, and the appropriateness of the paired statistical analysis, were additionally considered. Disagreements between the reviewers were resolved by consensus or consultation with a third reviewer.

The certainty of evidence for each critical outcome was assessed using the Grading of Recommendations Assessment, Development and Evaluation (GRADE) approach. Evidence from randomized trials initially received a high-certainty rating and was downgraded by one or two levels for serious or very serious concerns, respectively, regarding risk of bias, inconsistency, indirectness, imprecision, and publication bias. Certainty was classified as high, moderate, low, or very low.

GRADE assessments were conducted separately for serum BDNF, plasma BDNF, pain intensity, Pain Catastrophizing Scale total score, pain-related disability, and working memory. Serum and plasma BDNF were considered separately because biological matrix and preanalytical procedures can materially affect measured BDNF concentrations. The combined serum–plasma BDNF estimate was therefore not used for the principal GRADE assessment.

For pain catastrophizing and pain-related disability, certainty was based on the validated total scores rather than estimates that combined total scores with their component subscales or domains. These measurements are statistically dependent and represent different scale ranges; consequently, their combination as independent observations was not considered appropriate for the Summary of Findings table. Working-memory findings were assessed narratively because the studies used different instruments and cognitive constructs, making a pooled raw mean difference difficult to interpret.

### 2.4. Data Extraction

Data extraction was performed independently using a standardized Microsoft Excel sheet. Three main categories of data were collected. First, we extracted the general and demographic characteristics of the studies, including the first author, publication year, country, study design, clinical population, sample size, age, sex distribution, and follow-up period. Second, detailed intervention characteristics were recorded, including the tDCS montage, electrode placement, current intensity, session duration, total number of sessions, treatment setting, and any accompanying intervention. Corresponding details of the sham procedure were also collected. The final category included outcome data required for the quantitative analysis. For continuous outcomes, the post-treatment mean, standard deviation, and total number of participants in each group were extracted. The primary analyses were based on post-treatment comparisons between active and sham tDCS rather than changes from baseline. For dichotomous outcomes, the number of participants experiencing the event and the total number assessed in each group were recorded. For BDNF, the biological sample type, assay method, reporting unit, and assessment timepoint were documented. Concentrations reported in pg/mL were converted to ng/mL by dividing both the mean and standard deviation by 1000. Serum and plasma BDNF were analyzed in separate subgroups. When standard deviations were not directly available, they were calculated from standard errors, confidence intervals, or other measures of dispersion using established formulas. Data derived from medians and interquartile ranges or extracted from figures were clearly identified and evaluated in sensitivity analyses. Values with unclear or unconfirmed measurement units were not included in an absolute mean-difference analysis.

### 2.5. Data Synthesis and Analysis

The meta-analysis was performed using Review Manager and OpenMeta[Analyst]. Continuous outcomes were summarized using the mean difference and 95% confidence interval when the studies reported results on the same scale. Standardized mean differences were considered when different instruments were used to evaluate the same underlying construct. When different instruments assessed the same underlying construct, standardized mean differences were used to account for differences in measurement scale, whereas clinically or conceptually distinct outcomes were analyzed separately rather than combined. Dichotomous outcomes were analyzed using risk ratios with corresponding 95% confidence intervals. For BDNF, serum and plasma measurements were treated as distinct biological matrices and synthesized separately as prespecified analyses. No combined serum-plasma estimate was used for the principal interpretation because absolute BDNF concentrations differ substantially between these matrices and are influenced by different pre-analytical characteristics.

Other outcomes were subgrouped according to the clinical domain or measurement instrument when appropriate. For crossover trials, quantitative data were included only when the paired within-participant structure could be appropriately incorporated into the effect estimate. When the information required for an appropriate paired analysis was unavailable, the study was retained in the qualitative synthesis but excluded from the corresponding quantitative meta-analysis.

For multi-arm trials with a shared sham group, eligible active groups were combined when clinically appropriate. When distinct active comparisons were retained separately, the shared sham sample was divided across comparisons to avoid double-counting. The same sham participants were therefore not entered at their full sample size more than once within the same meta-analysis

Statistical heterogeneity was evaluated using the Chi-square test and the I^2^ statistic. A heterogeneity-test *p* value below 0.10 or an I^2^ value greater than 50% was considered indicative of substantial inconsistency among the included studies. Statistical significance for the pooled intervention effect was defined as a two-sided *p* value below 0.05. Leave-one-out sensitivity analyses were conducted by sequentially excluding one study at a time and repeating the meta-analysis. Additional sensitivity analyses excluded studies with indirectly estimated summary statistics, graph-derived data, unclear measurement units, crossover designs that could not be analyzed using paired data, or other relevant methodological concerns. The stability of the findings was assessed according to changes in the pooled effect estimate, confidence interval, statistical significance, and heterogeneity [26].

## 3. Results

### 3.1. Summary of Included Studies

**Figure 1:**
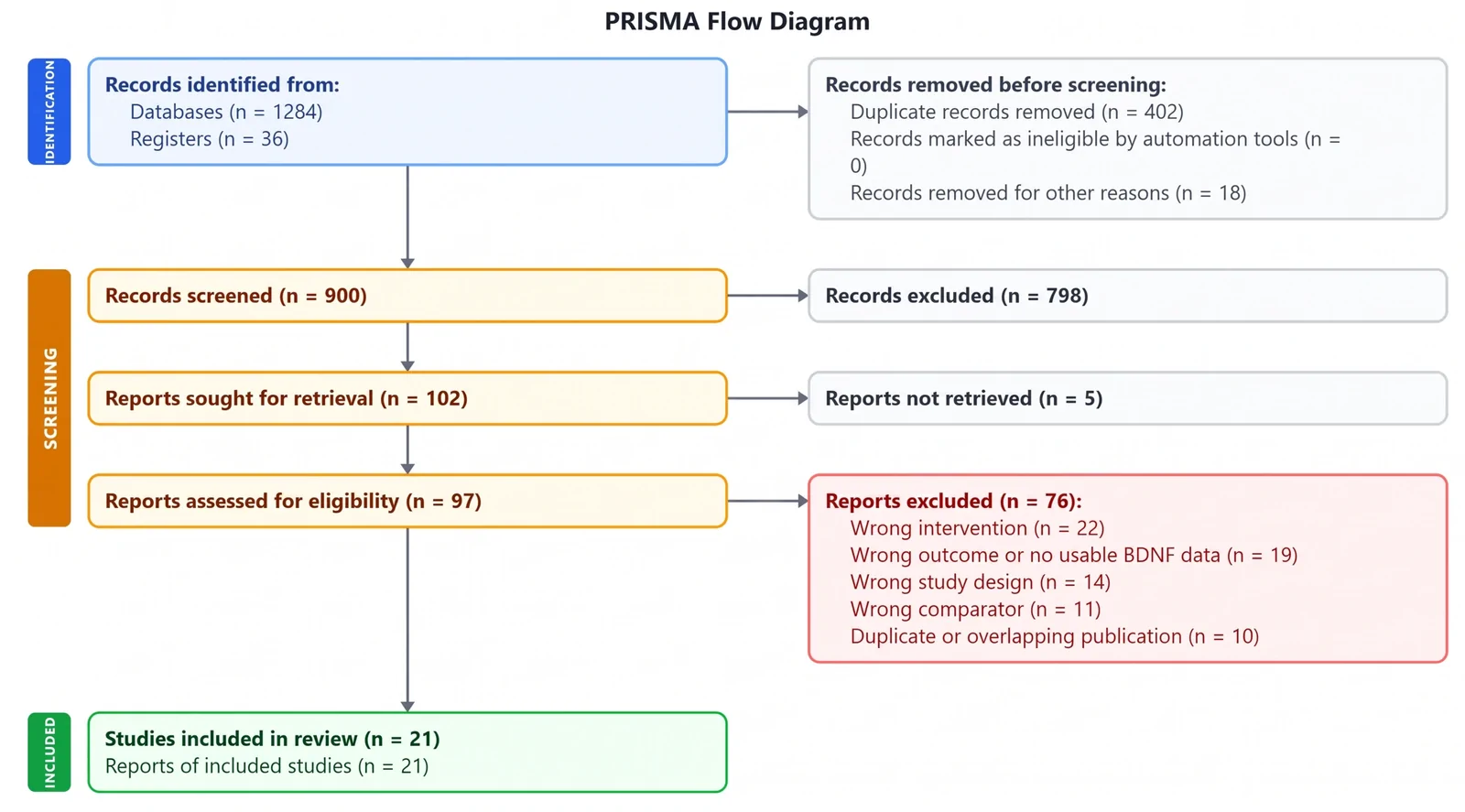
PRISMA flow diagram showing study identification, screening, eligibility assessment, and final inclusion.

The study selection process is presented in the PRISMA flow diagram (Figure 1). A total of 21 eligible study reports were included in the systematic review, of which 15 contributed to at least one quantitative synthesis. Studies that provided sufficient and clinically comparable post-treatment data were included in one or more quantitative meta-analyses, whereas reports with unavailable, incomplete, or unsuitable numerical data were retained in the qualitative synthesis only. Consequently, the number of studies and participants contributing to each pooled analysis varied according to the outcome assessed. The included studies compared active transcranial direct current stimulation with sham stimulation, either as a standalone intervention or alongside balanced co-interventions, including cognitive stimulation, aerobic exercise, physical therapy, gait training, working-memory training, and placebo-controlled pharmacological treatment. The assessed outcomes included circulating BDNF, inflammatory and neuroplasticity biomarkers, pain intensity, pain catastrophizing, pain-related disability and functional interference, and working memory. The demographic, clinical, and intervention characteristics of all included study reports are summarized in Table 1.

### 3.2. Results of the Quality Assessment & Certainty of Evidence

**Figure 2:**
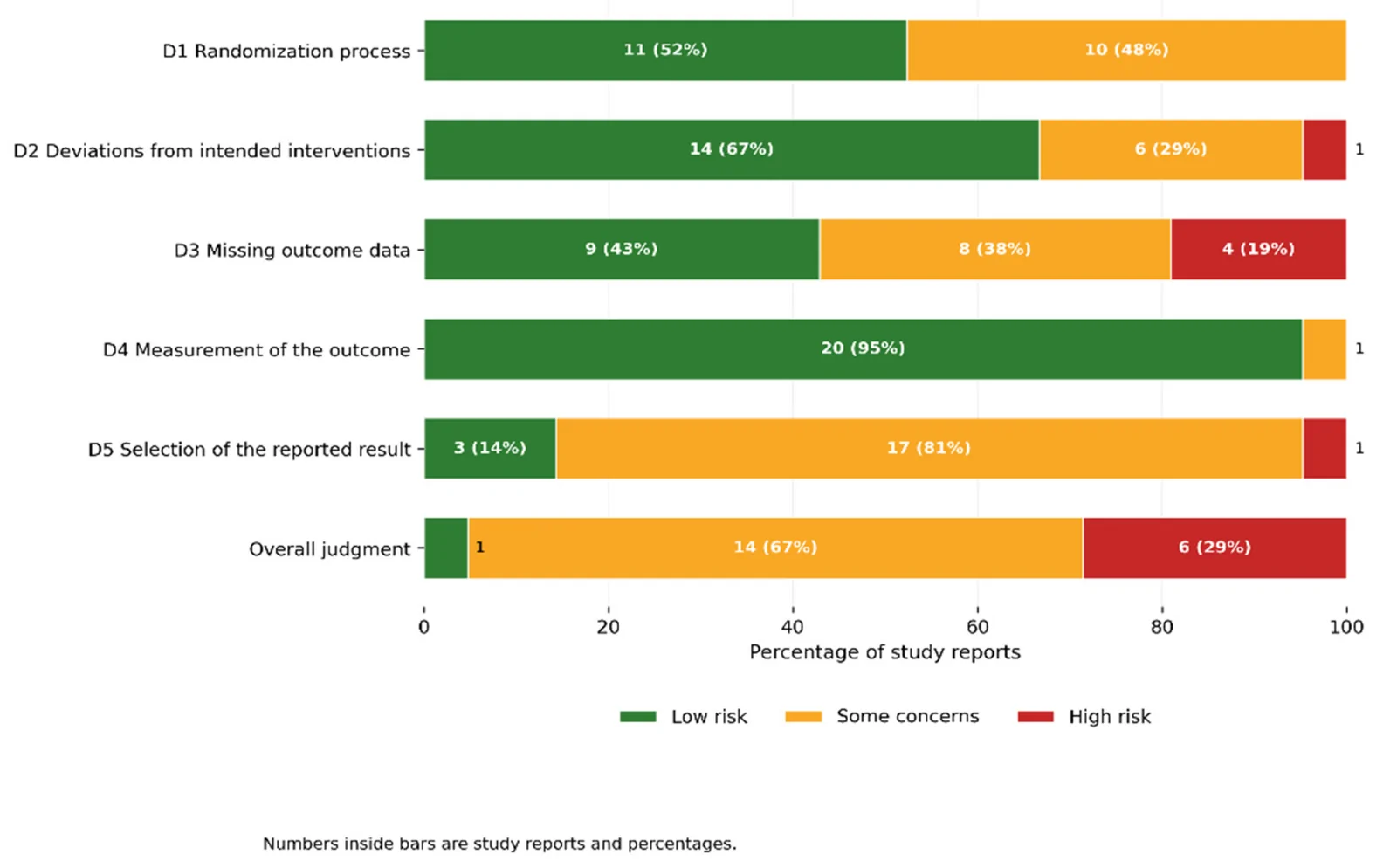
Summary of RoB 2 judgments across 21 study reports.

Twenty-one randomized study reports were assessed separately using the revised Cochrane risk-of-bias tool for randomized trials, with Serrano et al. and Caumo et al. evaluated as separate studies. For bias arising from the randomization process, 11 studies were judged as low risk and 10 as having some concerns. Bias due to deviations from the intended interventions was judged as low risk in 14 studies, as having some concerns in 6 studies, and as high risk in 1 study. For bias due to missing outcome data, 9 studies were judged as low risk, 8 as having some concerns, and 4 as high risk. Bias in measurement of the outcome was judged as low risk in 20 studies and as having some concerns in 1 study. For bias in selection of the reported result, 3 studies were judged as low risk, 17 as having some concerns, and 1 as high risk. Overall, 1 study was judged as having a low risk of bias, 14 as having some concerns, and 6 as having a high risk of bias. The main concerns involved substantial attrition and per-protocol analysis in Borzooee et al., post-randomization exclusion of sham participants in Alcalá-Lozano et al., exclusion of outcome outliers in Adam et al., incomplete BDNF measurements in SELECT-TDCS, and missing BDNF data with last-observation-carried-forward analysis in Palm et al. The complete assessment is presented in Figure 2 and Supplementary Table S1.

The certainty of evidence was rated as low for serum BDNF, plasma BDNF, pain intensity, the Pain Catastrophizing Scale total score, and the PCP:S total score. The certainty of evidence for working memory was rated as very low.

Serum and plasma BDNF evidence was downgraded for risk of bias and imprecision. Important limitations included incomplete biomarker measurements, per-protocol analyses, post-randomization exclusions, crossover concerns, and selective reporting of ancillary biomarker analyses. The pooled estimates included both possible increases and decreases in BDNF and did not establish a clinically meaningful treatment effect. The meta-analysis found no significant effect on serum BDNF or plasma BDNF.

Pain-intensity evidence was downgraded for risk of bias and inconsistency. Active tDCS reduced pain intensity compared with sham stimulation, but substantial unexplained heterogeneity was present across the three comparisons, with I^2^ = 82%.

Evidence for the Pain Catastrophizing Scale total score was downgraded for risk of bias and imprecision. Although the point estimate favored active tDCS, the confidence interval crossed the line of no effect. The statistically significant “overall” catastrophizing estimate was not used in the GRADE assessment because it combined PCS total scores with the helplessness, magnification, and rumination subscales from overlapping participants.

Evidence for pain-related disability measured using the PCP:S total score was downgraded for risk of bias and limited information size. The estimate favored active tDCS, but the result was based on a small number of comparisons. The broader estimate combining the PCP:S total score with emotional interference, activity interference, and pain-frequency domains was not used for GRADE because these were correlated measurements from the same participants.

Working-memory evidence was rated as very low because of risk of bias, substantial variation in the cognitive constructs and instruments used, and imprecision. Although the current analysis reported no significant effect and low statistical heterogeneity, the pooled raw mean difference is not clinically interpretable when measurements use different scales. The complete assessment is presented in Supplementary Table S2.

### 3.3. Analysis of Outcomes

#### 3.3.1. Brain-Derived Neurotrophic Factor

BDNF levels were assessed in twelve study comparisons. Serum and plasma BDNF were analyzed separately because they represent biologically and analytically distinct matrices. No significant difference was observed between active and sham tDCS for serum BDNF (MD = −0.01 [−0.15, 0.13], *p* = 0.890; I^2^ = 60%) or plasma BDNF (MD = 0.02 [−0.25, 0.29], *p* = 0.870; I^2^ = 35%). No combined serum-plasma estimate was used for physiological interpretation (Figure S1).

#### 3.3.2. Pain Intensity

Pain intensity was reported by three study comparisons. Active tDCS was associated with lower pain intensity than sham tDCS (MD = −1.79 [−2.34, −1.23], *p* < 0.00001). However, the estimated analysis was heterogeneous (*p* = 0.004, I^2^ = 82%) (Figure S2).

#### 3.3.3. Pain Catastrophizing

Regarding pain catastrophizing, three study comparisons assessed the PCS total score, while two comparisons assessed each of the helplessness, magnification, and rumination subscales. No significant difference was found between active and sham tDCS for the PCS total score (MD = −5.11 [−10.33, 0.12], *p* = 0.060), helplessness (MD = −0.98 [−4.15, 2.20], *p* = 0.550), magnification (MD = −0.71 [−2.93, 1.50], *p* = 0.530), or rumination (MD = −0.90 [−2.63, 0.83], *p* = 0.310). Although all point estimates were directionally favorable to active tDCS, the available evidence did not demonstrate a statistically significant effect on pain catastrophizing (Figure S3).

#### 3.3.4. Pain Disability and Functional Interference

Pain-related disability and functional interference were evaluated using the PCP:S total score and its component domains. Active tDCS was associated with a lower PCP:S total score than sham tDCS (MD = −11.39 [−16.67, −6.12], *p* < 0.0001) and lower emotional interference or burden (MD = −3.21 [−6.02, −0.41], *p* = 0.020). No statistically significant differences were observed for interference with daily activities (MD = −3.27 [−8.83, 2.30], *p* = 0.250) or pain frequency (MD = 0.47 [−3.54, 4.48], *p* = 0.820). The PCP:S total score and its component domains were interpreted separately because they represent correlated measurements derived from overlapping participants and were therefore not combined into a single overall pooled estimate (Figure S4).

#### 3.3.5. Working Memory

Working-memory performance was examined in seven study comparisons. There was no significant difference between active and sham tDCS in core working-memory measures (MD = 0.74 [−0.11, 1.60], *p* = 0.090) or secondary and supportive measures (MD = 0.11 [−0.09, 0.31], *p* = 0.270). The overall analysis also showed no significant difference between the two groups (MD = 0.14 [−0.05, 0.34], *p* = 0.150). The estimated analysis was homogeneous (*p* = 0.320, I^2^ = 14%) (Figure S5).

#### 3.3.6. Interleukins

The effects of active tDCS on interleukin levels were examined across the available study comparisons. No significant differences were observed for IL-6 (MD = 2.10 [−0.46, 4.66], *p* = 0.110), IL-10 (MD = −1.03 [−3.29, 1.23], *p* = 0.370), IL-1 (MD = 0.55 [−0.27, 1.37], *p* = 0.190), IL-8 (MD = −0.30 [−3.01, 2.42], *p* = 0.830), or IL-12p70 (MD = 0.22 [−0.18, 0.63], *p* = 0.280). Because these interleukins represent distinct biological analytes, they were interpreted separately rather than combined into a single overall pooled estimate (Figure S6).

#### 3.3.7. TNF-α and GDNF

Six study comparisons provided data for TNF-α and GDNF. Active tDCS did not significantly affect TNF-α levels compared with sham stimulation (MD = 0.08 [−0.31, 0.47], *p* = 0.680). Similarly, no significant difference was detected in GDNF levels (MD = 4.21 [−11.05, 19.46], *p* = 0.590). TNF-α and GDNF were interpreted separately because they represent biologically distinct biomarkers and were not combined into a single overall pooled estimate (Figure S7).

#### 3.3.8. Interleukin-18

For IL-18, two study comparisons were available. There was no significant difference between active and sham tDCS (MD = 3.59 [−16.50, 23.67], *p* = 0.730). The estimated analysis was homogeneous (*p* = 0.970, I^2^ = 0%) (Figure S8).

#### 3.3.9. Soluble Tumor Necrosis Factor Receptors

Soluble tumor necrosis factor receptors were evaluated in four study comparisons. No significant difference was found between active and sham tDCS for sTNFR1 (MD = 1.20 [−111.61, 114.01], *p* = 0.980) or sTNFR2 (MD = 39.14 [−277.89, 356.17], *p* = 0.810). The two receptor subtypes were interpreted separately rather than combined into a single overall pooled estimate (Figure S9).

## 4. Discussion

Our synthesis found no significant difference between active and sham tDCS in circulating serum or plasma BDNF. No statistically significant differences were also observed for working memory, pain catastrophizing as measured by the PCS total score or its individual subscales, interleukins, TNF-α, GDNF, IL-18, or soluble tumor necrosis factor receptors. For pain-related disability and functional interference, active tDCS was associated with lower PCP:S total scores and lower emotional interference or burden, whereas no significant differences were observed for interference with daily activities or pain frequency. Pain-intensity findings are discussed separately below in view of the small number of contributing studies and substantial heterogeneity. Serum and plasma BDNF were interpreted separately because these matrices differ substantially in their biological composition and pre-analytical characteristics.

The absence of a significant effect of active tDCS in either the serum or plasma BDNF analysis is consistent with prior meta-analysis that pooled peripheral BDNF data after non-invasive brain stimulation. Brunoni et al. (2015) examined eight datasets (*n* = 259) from patients with major depressive disorder receiving tDCS or repetitive transcranial magnetic stimulation and reported an essentially zero effect size (Hedges’ g = 0.03, 95% CI −0.21 to 0.27), with no change when tDCS was analyzed separately [29]. A subsequent meta-analysis restricted to repetitive transcranial magnetic stimulation similarly found no increase in serum BDNF (Hedges’ g = −0.12, 95% CI −0.30 to 0.06) [30]. An umbrella review of eleven meta-analyses in major depressive disorder further concluded that peripheral BDNF concentrations did not change after non-pharmacological interventions, including brain stimulation and physical activity, in contrast to the increases observed with pharmacological antidepressants [31]. Individual sham-controlled trials in treatment-resistant depression, bipolar depression, and chronic aphasia have likewise reported no significant between-group differences in serum or plasma BDNF after tDCS [32,33,34]. The present analysis extends previous evidence by synthesizing sham-controlled tDCS studies across a broader range of clinical populations and by evaluating serum and plasma BDNF separately.

A key consideration is the dissociation between central and peripheral BDNF. Rodent studies consistently demonstrate that anodal tDCS increases BDNF expression in stimulated brain regions, including the hippocampus and prefrontal cortex, and that these effects are dependent on tropomyosin receptor kinase B signaling [10,11,12]. A systematic review and meta-analysis of rodent and human data confirmed significant BDNF upregulation in stimulated brain tissue (*p* < 0.005) but did not pool human peripheral BDNF data [35]. This central–peripheral dissociation may reflect several factors: the blood–brain barrier limits bidirectional BDNF transport; the majority of serum BDNF originates from megakaryocytes and platelets rather than from the brain; tDCS may induce region-specific and even directionally opposing BDNF changes across the cerebral cortex, brainstem, and spinal cord that cancel out when measured peripherally; and the timing of blood sampling relative to stimulation may miss transient peaks or troughs [35]. Collectively, these observations suggest that peripheral BDNF may not be a reliable surrogate for tDCS-induced central neuroplasticity.

The reduction in pain intensity observed in the present analysis should be considered in terms of both statistical and clinical relevance. Prior meta-analytic evidence has also reported modest analgesic effects of tDCS. O’Connell et al. (2018) pooled 27 trials (*n* = 747) and reported an SMD of −0.43 (95% CI −0.63 to −0.22), corresponding to approximately 0.82 points on a 0–10 pain scale, while Wen et al. (2022) reported a similar short-term effect (SMD = −0.43, 95% CI −0.75 to −0.12) [36,37].

In the rheumatoid arthritis trial by Pilotti et al. (2026), an MCID of 1.1 points on the 0–10 VAS was prespecified [22]. Broader chronic-pain evidence using an 11-point numerical pain rating scale has suggested that a reduction of approximately 2 points or 30% from baseline corresponds to clinically important improvement at the individual-patient level [38]. However, such thresholds primarily describe within-patient improvement and should not be directly equated with a pooled between-group post-treatment mean difference. The clinical importance of between-group differences requires consideration of the magnitude and precision of the effect together with responder outcomes and other clinical factors [39].

The substantial heterogeneity in the pain-intensity analysis may also reflect important differences in pain pathophysiology and stimulation target across the included trials. Two comparisons involved fibromyalgia, in which altered central sensory processing, central sensitization, impaired descending pain modulation, and cognitive-affective amplification of pain are prominent, whereas Pilotti et al. evaluated patients with rheumatoid arthritis who had persistent pain despite low inflammatory activity, a setting in which peripheral inflammatory mechanisms may coexist with or give way to central sensitization and impaired descending inhibition. The cortical target may therefore be relevant to the mechanism of analgesia. M1 stimulation is principally intended to modulate sensorimotor and thalamocortical pain-processing networks and strengthen descending inhibitory control, whereas DLPFC stimulation may exert greater effects on cognitive-affective pain processing, attention, expectations, and top-down modulation of pain.

This distinction is relevant because the included fibromyalgia studies used both M1- and DLPFC-targeted protocols, while the rheumatoid arthritis trial targeted M1. Differences in stimulation schedule and concomitant treatment further increase clinical and mechanistic heterogeneity. Consequently, the pooled pain estimate should be interpreted as an average across distinct pain mechanisms and neuromodulatory strategies rather than as evidence of a uniform analgesic effect of tDCS across chronic pain conditions. More broadly, the pooled BDNF estimates should be interpreted as average effects across clinically and methodologically heterogeneous settings rather than as a single biological effect applicable uniformly across populations.

Differences in underlying disease, baseline pathophysiology, stimulation target and montage, current intensity, treatment schedule, concomitant interventions, and biomarker sampling may modify the peripheral BDNF response. Because most disease- and protocol-specific categories were represented by only one or a few comparisons, reliable subgroup estimates could not be generated. This limits the biological coherence and generalizability of the pooled estimates and should be considered when interpreting the absence of a consistent serum or plasma BDNF effect.

The favorable overall effect on pain catastrophizing (MD = −1.41, *p* = 0.040) appears to represent one of the first pooled meta-analytic estimates for this outcome. Prior evidence has been limited to individual trials, such as the finding by Caumo et al. (2022) that home-based bifrontal tDCS reduced Pain Catastrophizing Scale scores by approximately 51% versus 27% with sham in fibromyalgia [40]. The observation that the overall effect reached significance while none of the individual subscales did suggests that the effect may be distributed across cognitive and affective dimensions of catastrophizing but is too small within any single domain to be detected with the available sample sizes.

The absence of significant effects on interleukins, TNF-α, GDNF, IL-18, and soluble tumor necrosis factor receptors is consistent with the largest available human trial data. In the ELECT-TDCS trial (*n* = 236), peripheral biomarkers including IL-1β, IL-6, IL-8, IL-10, IL-12p70, IL-18, TNF-α, sTNFR1, and sTNFR2 were not associated with tDCS treatment outcome after correction for multiple comparisons; several cytokines decreased over time regardless of group allocation [20]. The BETTER trial in bipolar depression (*n* = 52) found a significant time-by-group interaction only for IL-8, with all other biomarkers showing no change [21]. Animal studies, by contrast, demonstrate that tDCS can modulate central neuroinflammation by shifting microglial and astrocytic polarization and suppressing the NF-κB/NLRP3/IL-18 pathway within brain tissue [41,42]. These central anti-inflammatory effects may not translate to detectable changes in peripheral blood, particularly given that tDCS delivers subthreshold electric fields (0.2–0.5 V/m) that modulate cortical excitability rather than systemic immune function [43,44]. A double-blind trial in 79 healthy males found that five sessions of tDCS induced no significant changes across 102 circulating metabolites, with power calculations indicating that sample sizes of up to 11,000 per group would be needed to detect the observed variation [45].

The biological effects of tDCS may also be captured through hemodynamic measures that complement circulating biomarkers. Straudi et al. (2024) investigated ten patients with prolonged traumatic disorders of consciousness in a minimally conscious state who received ten sessions of bilateral M1 anodal tDCS. Functional near-infrared spectroscopy (fNIRS) demonstrated a significant increase in cortical hemodynamic activation after a single stimulation session, while repeated stimulation was associated with reductions in angiopoietin-2, VEGF-C, and IP-10; changes in inflammatory markers were also correlated with behavioral changes measured using the Coma Recovery Scale-Revised [46]. Although exploratory and based on a small sample, these findings illustrate that tDCS-related biological responses may involve neurovascular and inflammatory mechanisms that are not adequately characterized by peripheral biomarkers alone. The integration of fNIRS or other functional neuroimaging approaches with circulating biomarkers may therefore provide a more comprehensive assessment of the neurobiological effects of tDCS, including in severe neurological conditions such as disorders of consciousness.

The null finding for working memory is also consistent with the broader literature. Meta-analytic evidence indicates that tDCS effects on working memory are small (effect sizes of 0.17–0.34); highly dependent on stimulation parameters, task type, and population; and often reduced to non-significance after correction for publication bias [47,48,49]. The included studies in the present analysis were not uniformly optimized for working-memory enhancement (e.g., online stimulation during training with left dorsolateral prefrontal cortex targeting), which may have contributed to the null result.

The dissociation between clinical pain improvement and the absence of peripheral biomarker changes can be understood through the predominantly central mechanisms of tDCS-mediated analgesia. Motor cortex stimulation activates descending pain inhibitory pathways through the periaqueductal gray, inhibits thalamic sensory neurons, and enhances conditioned pain modulation [50,51,52,53]. Positron emission tomography evidence indicates that motor cortex tDCS activates μ-opioid receptor neurotransmission in the periaqueductal gray, precuneus, thalamus, and prefrontal cortex [50]. These top-down modulatory effects operate through changes in cortical excitability, synaptic plasticity, and neurotransmitter release that are not obligately reflected in peripheral blood biomarkers.

### Limitations

Several limitations should be acknowledged. First, the number of studies contributing to each outcome was limited, particularly for pain catastrophizing, IL-18, and soluble tumor necrosis factor receptors, reducing statistical power and the ability to detect small effects.

Second, substantial clinical heterogeneity was present across the included trials regarding the underlying condition, stimulation protocol, electrode montage, current intensity, session number, co-interventions, and timing of outcome assessment. The broad inclusion of healthy participants and patients with neurologic, psychiatric, and chronic pain conditions introduced clinical heterogeneity and might have obscured diagnosis-specific effects. Diagnosis-stratified analyses were not considered sufficiently reliable because most conditions were represented by only one or a small number of BDNF comparisons. Also, the pain-intensity analysis was based on only three study comparisons, substantially limiting the precision and robustness of the pooled estimate. Because so few studies were available, small-study effects and publication bias cannot be reliably assessed and therefore remain possible sources of uncertainty. Accordingly, the pain findings should be considered preliminary until replicated in a larger body of randomized evidence. This heterogeneity was reflected in high I^2^ values for several pain-related and inflammatory biomarker analyses.

Third, BDNF measurement varied across studies in terms of the biological sample (serum versus plasma), assay method, reporting units, and blood collection procedures, all of which can substantially influence reported concentrations.

Fourth, most included trials had small sample sizes, and several were assessed as having some concerns or high risk of bias, particularly regarding blinding integrity and selective outcome reporting.

Fifth, the analyses were based on post-treatment comparisons rather than change-from-baseline data, which may not fully account for baseline imbalances.

Sixth, some summary statistics were estimated from medians, interquartile ranges, or graphical data, introducing additional imprecision.

Seventh, although differences in measurement scales were addressed using standardized mean differences when appropriate, residual heterogeneity may remain because clinical instruments differ in sensitivity, responsiveness, and construct coverage, while laboratory assays differ in analytical sensitivity, sample processing, storage, and other pre-analytical characteristics.

Finally, the timing of blood sampling relative to the last stimulation session was inconsistent across studies, and transient biomarker changes may have been missed. Future trials should standardize BDNF measurement procedures, including the biological sample, clotting duration, centrifugation protocol, storage conditions, and assay type, and should report whether the assay distinguishes mature BDNF from pro-BDNF. Serial blood sampling at multiple timepoints after stimulation would help characterize the temporal dynamics of any biomarker response. Larger, adequately powered trials with pre-registered primary outcomes are needed, particularly for pain catastrophizing and disability, where the present analysis suggests potential effects that require confirmation. Stratification by BDNF Val66Met polymorphism status, baseline biomarker levels, and clinical population would help identify subgroups that may respond differently.

Future studies should adopt multimodal approaches that integrate clinical outcomes with peripheral biomarkers, neurophysiological measures, and neuroimaging techniques to better characterize tDCS-induced neuroplasticity and determine how central biological responses relate to clinically meaningful changes across different patient populations.

The cerebellum may also represent an important target for future neuromodulation studies. Beyond its established role in motor control, it participates in sensorimotor integration, cognitive-affective processing, and pain modulation through extensive cerebello-cortical and cerebello-subcortical networks, and its involvement has increasingly been recognized across chronic pain and neurological disorders [54,55]. Future studies should therefore investigate whether cerebellar tDCS, including network-informed targeting of specific cerebellar regions, can modulate clinical outcomes and neuroplasticity-related responses.

Finally, longer follow-up periods are needed to determine whether the observed pain benefits are sustained beyond the immediate post-treatment period, given that prior evidence suggests attenuation of effects beyond six weeks.

## 5. Conclusions

This systematic review and meta-analysis of 21 studies found that active tDCS did not produce significant changes in circulating serum or plasma BDNF compared with sham stimulation, a finding consistent with prior pooled analyses and individual trials across multiple clinical populations. Similarly, no significant effects were observed for interleukins, TNF-α, GDNF, IL-18, soluble tumor necrosis factor receptors, or working memory. In contrast, active tDCS was associated with significant reductions in pain intensity, a favorable overall effect on pain catastrophizing, and improvements in pain-related disability and functional interference, including the emotional interference domain.

The absence of peripheral biomarker changes should not be interpreted as evidence of absent central neuroplastic effects, as circulating BDNF and other peripheral measures may not adequately reflect localized or transient biological changes within the central nervous system. Although active tDCS was associated with improvements in several pain-related outcomes, the limited number of contributing studies, substantial heterogeneity, and low certainty of the evidence preclude firm conclusions regarding its clinical effectiveness for pain management. Larger, methodologically rigorous trials with standardized biomarker protocols, serial sampling, and longer follow-up are needed to confirm these clinical findings and clarify the biological mechanisms underlying them.

## Figures and Tables

**Table 1:** Characteristics of Included Studies.

Study	Country	Population	Design	Montage/Target	Anode	Cathode	Current (mA)	Session Duration (min)	Total Sessions	Treatment Period	Co-Intervention	BDNF Specimen	Assay	Assessment Timepoints
Palm et al., 2013	Germany	Treatment-resistant major depression	Double-blind crossover RCT	F3–right supraorbital	F3	Right supraorbital	1 or 2	20	10 per period	Per treatment period	Stable medication	Serum	ELISA	Baseline, week 2, week 4
Brunoni et al., 2018 (ELECT-TDCS)	Brazil	Unipolar major depression	Randomized non-inferiority RCT	Bifrontal DLPFC	F3	F4	2	30	22	10 weeks	Placebo pill	Plasma	ELISA	Baseline, week 3, week 10
Ji et al., 2023	China	Healthy young adults	Three-arm RCT	Left DLPFC–FP2	F3	FP2	2	30	20	4 weeks	Aerobic exercise	Serum	ELISA	Pre-intervention, post-intervention
Youssef et al., 2023	Egypt	Subacute MCA ischemic stroke	Participant-blind three-arm RCT	M1 montages	Ipsilesional M1	Contralesional M1 or supraorbital	2	20	12	4 weeks	45-min physical therapy in all groups	Serum	ELISA	Pre-intervention, post-intervention
Borzooee et al., 2024	Iran	Male OUD patients on maintenance treatment	Triple-blind sham-controlled RCT	Right DLPFC–FP1	F4	FP1	2	13	10	5 days	Methadone, buprenorphine, or opium tincture	Serum	Sandwich ELISA	Baseline, day 5
Kazemzadeh et al., 2026	Iran	Male OUD patients on methadone	Secondary machine-learning analysis	Bilateral DLPFC with reversed polarity	NR	NR	NR	NR	NR	NR	Methadone	Blood	NR	Pre-intervention, post-intervention
Eskandari et al., 2019	Iran	Male opioid-addicted patients	Randomized three-arm pre/post study	Bilateral DLPFC	NR	NR	NR	20	10	NR	Conventional treatment	Serum	ELISA	Pre-intervention, post-intervention
Adam et al., 2021	France	Schizophrenia with resistant hallucinations	Double-blind sham-controlled RCT	Frontotemporal	Left prefrontal cortex	Left temporoparietal junction	2	20	1	Single session	NR	Serum	ELISA	Immediately before, immediately after
Serrano et al., 2022	Brazil	Women with fibromyalgia	Double-blind sham-controlled RCT	Bifrontal DLPFC	F3	F4	2	20	20	4 weeks	NR	Serum	Sandwich ELISA	Baseline, treatment end
Brietzke et al., 2016	Brazil	Chronic hepatitis C with treatment-related pain	Phase II double-blind RCT	Left M1–right supraorbital	Left M1	Right supraorbital	2	20	5	5 days	Standard hepatitis C treatment	Serum	ELISA	Baseline, treatment end
Goerigk et al., 2021 (BETTER)	Brazil	Bipolar depression	Exploratory biomarker analysis of RCT	Bifrontal DLPFC	NR	NR	2	30	12	6 weeks	Stable pharmacotherapy	Plasma	ELISA	Baseline, week 6
Alcalá-Lozano et al., 2025	Mexico	Amnestic mild cognitive impairment	Double-blind placebo-controlled RCT	Bifrontal DLPFC	F3	F4	2	30	15	3 weeks	9 cognitive-stimulation sessions in both groups	Serum	ELISA	T0, T1
Beltran Serrano et al., 2020	Brazil	Healthy women	Randomized incomplete crossover study	Bifrontal DLPFC	F3	F4	2	20	Single sessions	≥7-day washout	Hypnotic suggestion where assigned	Serum	NR	Baseline only
de Paula et al., 2023	Brazil	Women with fibromyalgia	Double-blind 2 × 2 placebo/sham RCT	M1–contralateral supraorbital	M1	Contralateral supraorbital	2	20	5	Last 5 days of 26-day drug period	LDN 4.5 mg or placebo for 26 days	Serum	ELISA	Day 1, pre-tDCS, post-tDCS
Marangolo et al., 2014	Italy	Chronic nonfluent post-stroke aphasia	Double-blind crossover study	Bihemispheric Broca areas	F5	F7	2	20	10 per condition	14-day interval	Language therapy	Serum	ELISA	Baseline, session 10, 1-week follow-up
Suchting et al., 2021	USA	Older adults with symptomatic knee osteoarthritis	Secondary analysis of sham-controlled RCT	M1–contralateral supraorbital	M1	Contralateral supraorbital	2	20	5	5 days	NR	Plasma	ELISA	Baseline, day 5
Brunoni et al., 2014 (SELECT-TDCS)	Brazil	Major depressive disorder	Factorial double-blind RCT	Bifrontal DLPFC	F3	F4	2	30	12	6 weeks	Sertraline 50 mg/day where assigned	Plasma	ELISA	Baseline, week 6
Brietzke et al., 2020	Brazil	Women with fibromyalgia	Double-blind sham-controlled RCT	Bifrontal DLPFC	Left DLPFC	Right DLPFC	2	30	60	12 weeks	NR	Serum	ELISA	Baseline, session 60
Caumo et al., 2022	Brazil	Women with fibromyalgia	Double-blind sham-controlled RCT	Bifrontal DLPFC	F3	F4	2	20	20	4 weeks	NR	Serum	ELISA	Baseline, treatment end
Pilotti et al., 2026	Brazil	Women with rheumatoid arthritis and persistent pain	Double-blind sham-controlled RCT	Left M1–Fp2	C3 / left M1	Fp2	2	20	20	4 weeks	Usual pharmacological treatment	Serum	ELISA	Baseline, day 30
da Cunha et al., 2024	Brazil	Chronic post-stroke patients	Assessor-blind sham-controlled RCT	Bihemispheric M1	Ipsilesional M1	Contralesional M1	2	30	10	2 weeks	Foot-drop stimulation and gait training in both groups	Serum	ELISA	Baseline, 24 h after session 10

Abbreviations: BDNF, brain-derived neurotrophic factor; BETTER, Bipolar Depression Electrical Treatment Trial; DLPFC, dorsolateral prefrontal cortex; ELECT-TDCS, Escitalopram versus Electrical Current Therapy for Treating Depression Clinical Study; ELISA, enzyme-linked immunosorbent assay; LDN, low-dose naltrexone; M1, primary motor cortex; MCA, middle cerebral artery; mA, milliampere; min, minutes; NR, not reported (NR indicates that the specific parameter was not reported in the available study report and was not inferred or imputed); OUD, opioid use disorder; RCT, randomized controlled trial; SELECT-TDCS, Sertraline versus Electrical Current Therapy for Treating Depression Clinical Study; T0, baseline assessment; T1, post-intervention assessment; tDCS, transcranial direct current stimulation; USA, United States of America.

## Data Availability

The original contributions presented in this study are included in the article/Supplementary Materials.

## Supplementary Materials

The following supporting information can be downloaded at: https://www.mdpi.com/article/10.3390/jcm15186992/s1, Figure S1. Forest plot comparing the effect of active tDCS versus sham stimulation on circulating brain-derived neurotrophic factor (BDNF) concentrations; Figure S2. Forest plot of the mean difference in pain intensity between active transcranial direct current stimulation (tDCS) and sham stimulation; Figure S3. Forest plot comparing the effect of active tDCS versus sham stimulation on pain catastrophizing; Figure S4. Forest plot illustrating the effect of active tDCS compared to sham stimulation on pain-related disability and functional interference; Figure S5. Forest plot of the mean difference in working-memory performance between active tDCS and sham stimulation, stratified into core working-memory measures and secondary or supportive measures; Figure S6. Forest plot comparing the effects of active tDCS and sham stimulation on circulating interleukin levels, subgrouped by IL-6, IL-10, IL-1, IL-8, and IL-12p70.; Figure S7. Forest plot comparing the mean difference in circulating levels of tumor necrosis factor-alpha (TNF-α) and glial cell line-derived neurotrophic factor (GDNF) between active tDCS and sham stimulation; Figure S8. Forest plot of the mean difference in interleukin-18 (IL-18) concentrations between active tDCS and sham stimulation; Figure S9. Forest plot depicting the effect of active tDCS versus sham stimulation on soluble tumor necrosis factor receptors, specifically sTNFR1 and sTNFR2; Table S1. Risk-of-bias assessment using the Cochrane RoB 2 tool. Table S2. GRADE Summary of Findings. Supplementary File S1. The PRISMA checklist. Reference [56] is cited in the Supplementary Materials.
