# Supplementary material for: Effects of Transcranial Direct Current Stimulation Versus Sham Stimulation on Brain-Derived Neurotrophic Factor and Clinical Outcomes: A Systematic Review and Meta-Analysis
Source: J Clin Med. 2026 Sep 9;15(18):6992. doi: 10.3390/jcm15186992 (PMC13607446; doi:10.3390/jcm15186992)
Supplement: Supplementary file 1 [file jcm-15-06992-s001.zip › jcm-4492348-supplementary.pdf]

Supplementary Figures

Figure S1. Forest plot comparing the effect of active tDCS versus sham stimulation on circulating brain-derived neurotrophic factor (BDNF) concentrations.

Study or Subgroup	Experimental Mean	Experimental SD	Experimental Total	Control Mean	Control SD	Control Total	Weight	Mean Difference IV, Random, 95% CI
**1.1.1 Serum BDNF, ng/mL**								
Alcalá-Lozano 2025	0.26996	0.01255	8	0.27023	0.00889	10	35.9%	-0.00 [-0.01, 0.01]
Caumo 2022	36.3	30	32	28.26	26.26	16	0.0%	8.04 [-8.50, 24.58]
de Paula 2023	5.3	6.1	22	5.1	5.9	21	0.1%	0.20 [-3.39, 3.79]
Eskandari 2019	6.63	5.39	10	2.37	0.87	5	0.1%	4.26 [0.83, 7.69]
Ji 2023	5.84	11.3	20	4.98	10.9	20	0.0%	0.86 [-6.02, 7.74]
Pilotti 2026	1.44652	0.5115	17	1.20722	0.5964	17	7.4%	0.24 [-0.13, 0.61]
Serrano 2022	31.39	18.32	24	58.53	48.61	12	0.0%	-27.14 [-55.60, 1.32]
Youssef 2023	0.28	0.14	24	0.39	0.09	6	29.2%	-0.11 [-0.20, -0.02]
**Subtotal (95% CI)**			**157**			**107**	**72.7%**	**-0.01 [-0.15, 0.13]**
Heterogeneity: Tau² = 0.01; Chi² = 17.50, df = 7 (P = 0.01); I² = 60%								
Test for overall effect: Z = 0.14 (P = 0.89)								
**1.1.2 Plasma BDNF, ng/mL**								
Brunoni ELECT 2018	2.969	2.60222	79	2.43	1.67185	56	2.3%	0.54 [-0.18, 1.26]
Brunoni SELECT 2014	1.588	0.297	15	1.502	0.653	19	8.9%	0.09 [-0.24, 0.42]
Goerigk 2021	5.23712	3.59245	26	4.75289	2.94889	26	0.4%	0.48 [-1.30, 2.27]
Suchting 2021	0.2209	0.2507	19	0.3847	0.4094	19	15.7%	-0.16 [-0.38, 0.05]
**Subtotal (95% CI)**			**139**			**120**	**27.3%**	**0.02 [-0.25, 0.29]**
Heterogeneity: Tau² = 0.03; Chi² = 4.62, df = 3 (P = 0.20); I² = 35%								
Test for overall effect: Z = 0.17 (P = 0.87)								
**Total (95% CI)**			**296**			**227**	**100.0%**	**-0.01 [-0.13, 0.10]**
Heterogeneity: Tau² = 0.01; Chi² = 22.38, df = 11 (P = 0.02); I² = 51%								
Test for overall effect: Z = 0.22 (P = 0.82)								
Test for subgroup differences: Chi² = 0.04, df = 1 (P = 0.83), I² = 0%								

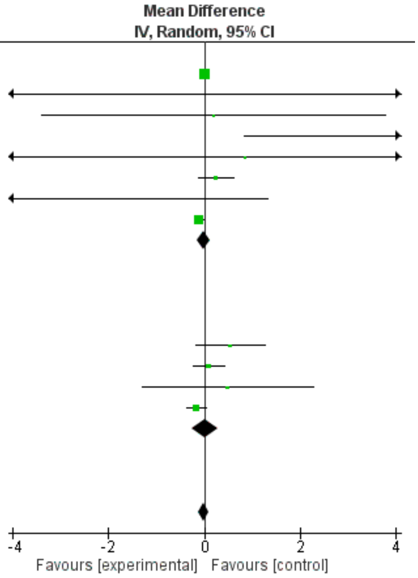

Figure S2. Forest plot of the mean difference in pain intensity between active transcranial direct current stimulation (tDCS) and sham stimulation.

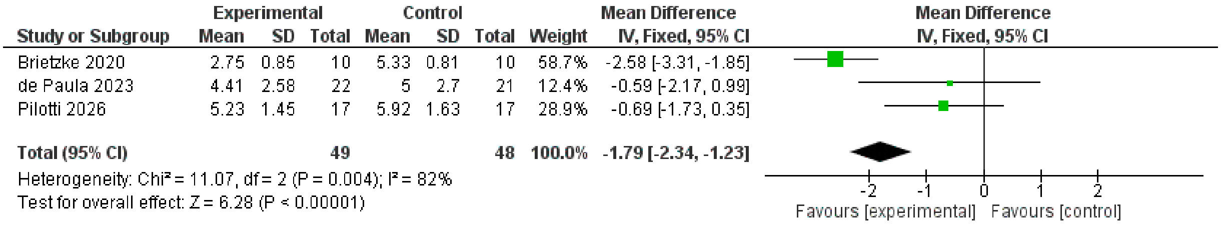

Figure S3. Forest plot comparing the effect of active tDCS versus sham stimulation on pain catastrophizing, including subgroup analyses for the Pain Catastrophizing Scale (PCS) total score and individual subscales (helplessness, magnification, and rumination).

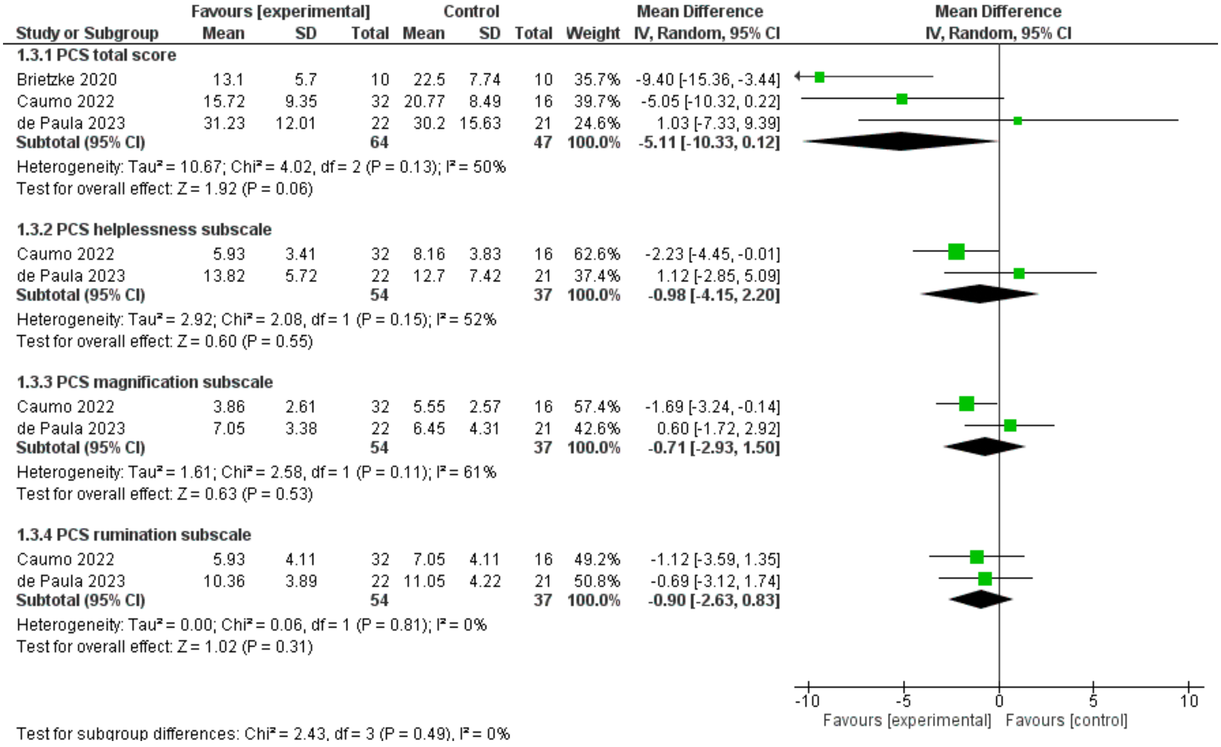

Figure S4. Forest plot illustrating the effect of active tDCS compared to sham stimulation on pain-related disability and functional interference, categorized by the Profile of Chronic Pain: Screen (PCP:S) total score, interference with daily activities, emotional interference or burden, and pain frequency.

	Favours [experimental]			Control				Std. Mean Difference
**Study or Subgroup**	**Mean**	**SD**	**Total**	**Mean**	**SD**	**Total**	**Weight**	**IV, Random, 95% CI**
**1.4.1 PCP:S total score**								
Brietzke 2020	36.88	5.76	10	49.78	8.74	10	23.8%	-1.67 [-2.72, -0.62]
Caumo 2022	47.2	17.23	32	54.53	19.09	16	39.7%	-0.40 [-1.01, 0.20]
Pilotti 2026	40	25.53	17	50.7	21.11	17	36.5%	-0.45 [-1.13, 0.24]
**Subtotal (95% CI)**			**59**			**43**	**100.0%**	**-0.72 [-1.38, -0.06]**
Heterogeneity: Tau² = 0.19; Chi² = 4.58, df = 2 (P = 0.10); I² = 56%								
Test for overall effect: Z = 2.14 (P = 0.03)								
**1.4.2 PCP:S interference with daily activities**								
Brietzke 2020	16.85	3.23	10	24.35	4.88	10	28.4%	-1.74 [-2.80, -0.67]
Caumo 2022	18.58	7.66	32	22.89	6.47	16	35.7%	-0.58 [-1.19, 0.03]
de Paula 2023	25	6.99	22	22.15	10.27	21	35.9%	0.32 [-0.28, 0.92]
**Subtotal (95% CI)**			**64**			**47**	**100.0%**	**-0.59 [-1.61, 0.44]**
Heterogeneity: Tau² = 0.66; Chi² = 11.84, df = 2 (P = 0.003); I² = 83%								
Test for overall effect: Z = 1.12 (P = 0.26)								
**1.4.3 PCP:S emotional interference/burden**								
Brietzke 2020	10.9	3.12	10	16.15	3.6	10	25.1%	-1.49 [-2.51, -0.48]
Caumo 2022	11.2	6.71	32	14.88	4.74	16	37.2%	-0.59 [-1.20, 0.02]
de Paula 2023	14.09	5.91	22	14.38	6.05	21	37.7%	-0.05 [-0.65, 0.55]
**Subtotal (95% CI)**			**64**			**47**	**100.0%**	**-0.61 [-1.33, 0.10]**
Heterogeneity: Tau² = 0.26; Chi² = 5.94, df = 2 (P = 0.05); I² = 66%								
Test for overall effect: Z = 1.68 (P = 0.09)								
**1.4.4 PCP:S pain frequency**								
Brietzke 2020	49.89	14.62	10	38.11	19.86	10	26.8%	0.65 [-0.26, 1.55]
Caumo 2022	17.65	4.94	32	19.98	5.49	16	36.6%	-0.45 [-1.05, 0.16]
de Paula 2023	25.77	2.16	22	24.3	4.35	21	36.7%	0.42 [-0.18, 1.03]
**Subtotal (95% CI)**			**64**			**47**	**100.0%**	**0.16 [-0.50, 0.83]**
Heterogeneity: Tau² = 0.22; Chi² = 5.62, df = 2 (P = 0.06); I² = 64%								
Test for overall effect: Z = 0.48 (P = 0.63)								

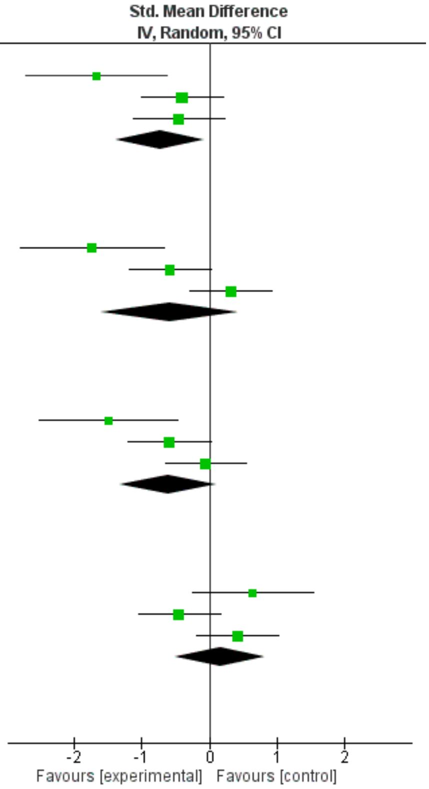

Test for subgroup differences: Chi² = 4.10, df = 3 (P = 0.25), I² = 26.9%

Figure S5. Forest plot of the mean difference in working-memory performance between active tDCS and sham stimulation, stratified into core working-memory measures and secondary or supportive measures.

Study or Subgroup	Experimental Mean	Experimental SD	Experimental Total	Control Mean	Control SD	Control Total	Weight	Mean Difference IV, Fixed, 95% CI
**1.5.1 Core working-memory measures**								
Alcalá-Lozano 2025	15.88	3.52	8	14.6	5.02	10	0.2%	1.28 [-2.67, 5.23]
Ji 2023	6.91	5.098	20	6.01	5.277	20	0.4%	0.90 [-2.32, 4.12]
Santos 2018	5.05	1.82	19	4.73	2.02	20	2.6%	0.32 [-0.89, 1.53]
Serrano 2022	6	2.65	24	4.8	1.54	12	2.0%	1.20 [-0.17, 2.57]
**Subtotal (95% CI)**			**71**			**62**	**5.2%**	**0.74 [-0.11, 1.60]**
Heterogeneity: Chi² = 0.98, df = 3 (P = 0.81); I² = 0%								
Test for overall effect: Z = 1.71 (P = 0.09)								
**1.5.2 Secondary or supportive measures**								
Ji 2023	0.95	0.134	20	0.88	0.447	20	89.4%	0.07 [-0.13, 0.27]
Santos 2018	6.45	1.5	19	6	1.69	20	3.7%	0.45 [-0.55, 1.45]
Serrano 2022	8.11	2.62	24	6.6	1.85	12	1.7%	1.51 [0.03, 2.99]
**Subtotal (95% CI)**			**63**			**52**	**94.8%**	**0.11 [-0.09, 0.31]**
Heterogeneity: Chi² = 4.02, df = 2 (P = 0.13); I² = 50%								
Test for overall effect: Z = 1.09 (P = 0.27)								
**Total (95% CI)**			**134**			**114**	**100.0%**	**0.14 [-0.05, 0.34]**
Heterogeneity: Chi² = 7.01, df = 6 (P = 0.32); I² = 14%								
Test for overall effect: Z = 1.45 (P = 0.15)								
Test for subgroup differences: Chi² = 2.01, df = 1 (P = 0.16), I² = 50.4%								

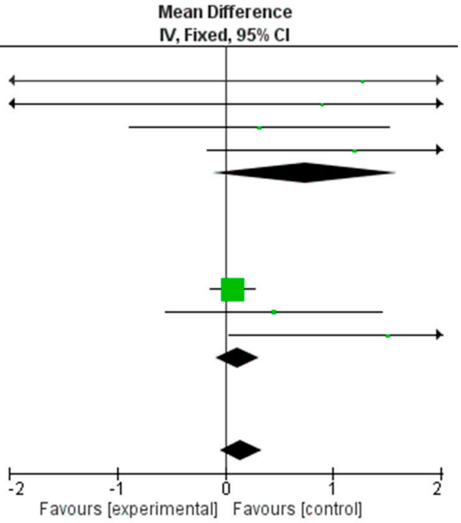

Figure S6. Forest plot comparing the effects of active tDCS and sham stimulation on circulating interleukin levels, subgrouped by IL-6, IL-10, IL-1, IL-8, and IL-12p70.

Study or Subgroup	Favours [experimental] Mean	SD	Total	Control Mean	SD	Total	Weight	Mean Difference IV, Random, 95% CI
**1.6.1 IL-6**								
Alcalá-Lozano 2025	17.59	3.86	24	17.78	5.83	12	29.3%	-0.19 [-3.83, 3.45]
Santos 2018	22.25	2.31	8	18.1	3.54	10	39.7%	4.15 [1.43, 6.87]
Serrano 2022	18.9	5.85	19	17.26	5.15	20	31.0%	1.64 [-1.83, 5.11]
**Subtotal (95% CI)**			**51**			**42**	**100.0%**	**2.10 [-0.46, 4.66]**
Heterogeneity: Tau² = 2.37; Chi² = 3.72, df = 2 (P = 0.16); I² = 46%								
Test for overall effect: Z = 1.61 (P = 0.11)								
**1.6.3 IL-10**								
Brunoni ELECT 2018	3.8	2.37	79	3.2	2.3	56	33.6%	0.60 [-0.20, 1.40]
da Cunha 2024	13.43	1.7	16	17.38	1.6	16	32.2%	-3.95 [-5.09, -2.81]
Goerigk 2021	2.16	1.19	26	2.05	1.02	26	34.2%	0.11 [-0.49, 0.71]
**Subtotal (95% CI)**			**121**			**98**	**100.0%**	**-1.03 [-3.29, 1.23]**
Heterogeneity: Tau² = 3.79; Chi² = 45.74, df = 2 (P < 0.00001); I² = 96%								
Test for overall effect: Z = 0.89 (P = 0.37)								
**1.6.4 IL-1**								
Brunoni ELECT 2018	6.4	8.44	79	6.2	5.63	56	12.0%	0.20 [-2.17, 2.57]
Goerigk 2021	3.9	1.73	26	3.29	2.03	16	47.1%	0.61 [-0.59, 1.81]
Pilotti 2026	1.56	2.6	17	0.98	0.9	26	40.9%	0.58 [-0.70, 1.86]
**Subtotal (95% CI)**			**122**			**98**	**100.0%**	**0.55 [-0.27, 1.37]**
Heterogeneity: Tau² = 0.00; Chi² = 0.10, df = 2 (P = 0.95); I² = 0%								
Test for overall effect: Z = 1.31 (P = 0.19)								
**1.6.6 IL-8**								
Brunoni ELECT 2018	7.3	6.96	79	6.1	6	56	46.1%	1.20 [-1.00, 3.40]
Goerigk 2021	8.04	2.56	26	9.62	3.22	26	53.9%	-1.58 [-3.16, 0.00]
**Subtotal (95% CI)**			**105**			**82**	**100.0%**	**-0.30 [-3.01, 2.42]**
Heterogeneity: Tau² = 2.91; Chi² = 4.05, df = 1 (P = 0.04); I² = 75%								
Test for overall effect: Z = 0.22 (P = 0.83)								
**1.6.7 IL-12p70**								
Brunoni ELECT 2018	4	2.74	79	3.4	2.15	56	23.1%	0.60 [-0.23, 1.43]
Goerigk 2021	2.17	0.8	26	2.06	0.79	26	76.9%	0.11 [-0.32, 0.54]
**Subtotal (95% CI)**			**105**			**82**	**100.0%**	**0.22 [-0.18, 0.63]**
Heterogeneity: Tau² = 0.01; Chi² = 1.06, df = 1 (P = 0.30); I² = 6%								
Test for overall effect: Z = 1.08 (P = 0.28)								

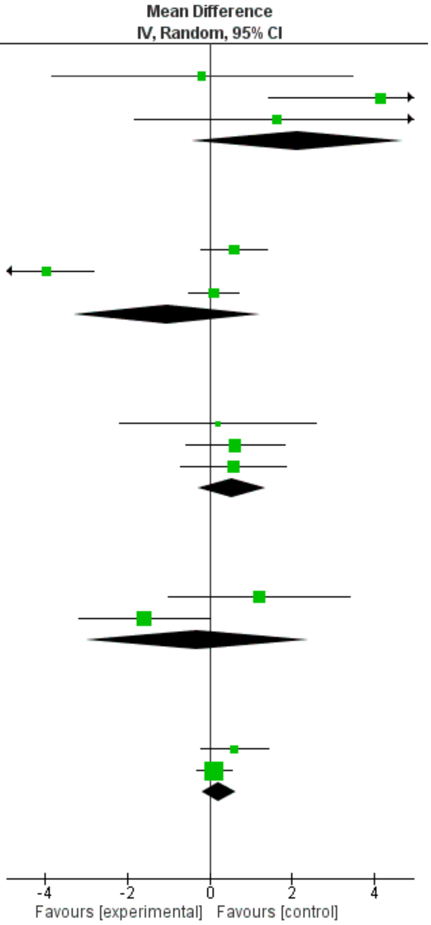

Test for subgroup differences: Chi² = 3.90, df = 4 (P = 0.42), I² = 0%

Figure S7. Forest plot comparing the mean difference in circulating levels of tumor necrosis factor-alpha (TNF-α) and glial cell line-derived neurotrophic factor (GDNF) between active tDCS and sham stimulation.

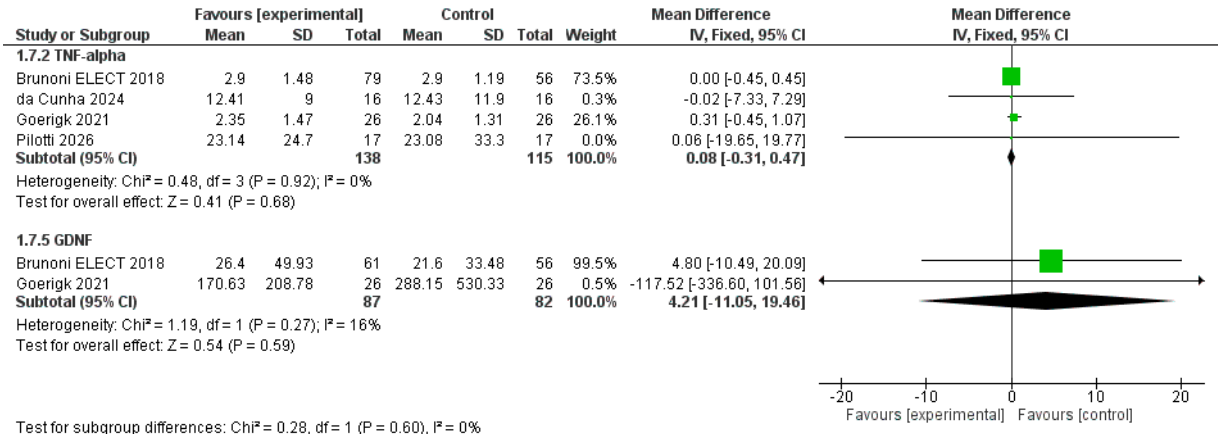

Figure S8. Forest plot of the mean difference in interleukin-18 (IL-18) concentrations between active tDCS and sham stimulation.

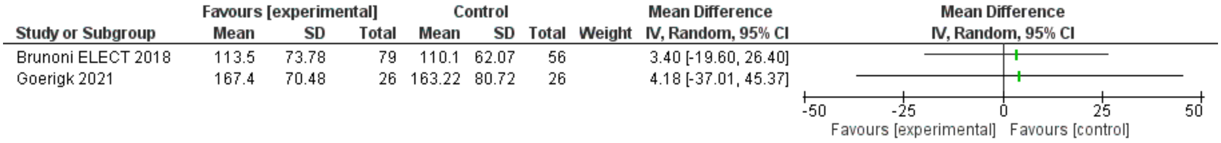

Figure S9. Forest plot depicting the effect of active tDCS versus sham stimulation on soluble tumor necrosis factor receptors, specifically sTNFR1 and sTNFR2.

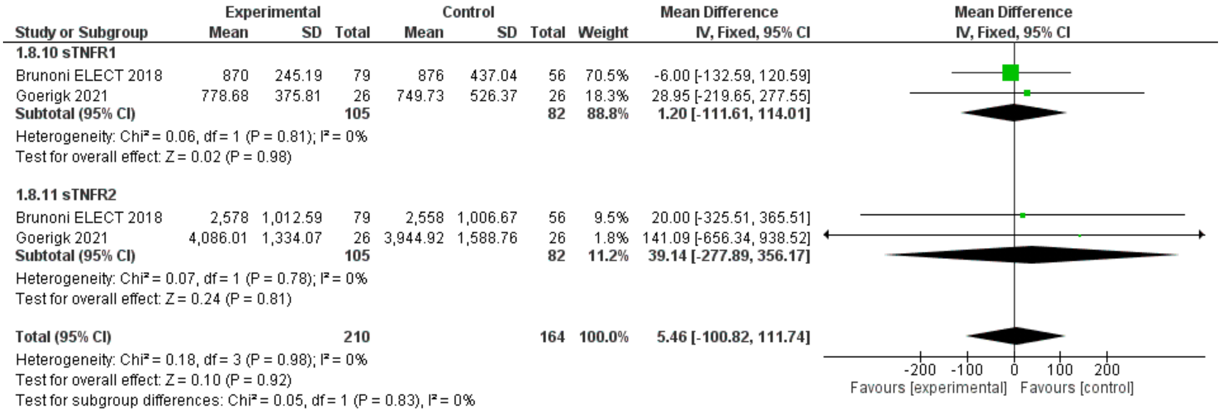

Supplementary Table S1. Risk-of-bias assessment using the Cochrane RoB 2 tool

Study	D1	D2	D3	D4	D5	Overall
**Palm et al., 2013**	Some concerns	High risk	Some concerns	Low risk	Some concerns	High risk
**Brunoni et al., 2018, ELECT-TDCS**	Low risk	Low risk	Some concerns	Low risk	Low risk	Some concerns
**Ji et al., 2023**	Some concerns	Low risk	Some concerns	Low risk	Some concerns	Some concerns
**Youssef et al., 2023**	Some concerns	Low risk	Low risk	Low risk	Some concerns	Some concerns
**Borzooee et al., 2024**	Some concerns	Low risk	High risk	Low risk	Some concerns	High risk
**Kazemzadeh et al., 2026**	Some concerns	Some concerns	Low risk	Some concerns	Some concerns	High risk
**Eskandari et al., 2019**	Some concerns	Some concerns	Low risk	Low risk	Some concerns	Some concerns
**Adam et al., 2021**	Some concerns	Low risk	High risk	Low risk	High risk	High risk
**Serrano et al., 2022**	Low risk	Some concerns	Some concerns	Low risk	Some concerns	Some concerns
**Brietzke et al., 2016**	Low risk	Low risk	Some concerns	Low risk	Low risk	Some concerns
**Goerigk et al., 2021, BETTER**	Low risk	Low risk	Some concerns	Low risk	Some concerns	Some concerns
**Alcalá-Lozano et al., 2025**	Some concerns	Low risk	High risk	Low risk	Some concerns	High risk
**Beltran Serrano et al., 2020**	Low risk	Some concerns	Low risk	Low risk	Some concerns	Some concerns
**de Paula et al., 2023**	Low risk	Low risk	Some concerns	Low risk	Some concerns	Some concerns
**Marangolo et al., 2014**	Some concerns	Some concerns	Low risk	Low risk	Some concerns	Some concerns
**Suchting et al., 2021**	Some concerns	Low risk	Low risk	Low risk	Some concerns	Some concerns

**Brunoni et al., 2014, SELECT-TDCS**	Low risk	Low risk	High risk	Low risk	Some concerns	High risk
**Brietzke et al., 2020**	Low risk	Low risk	Low risk	Low risk	Low risk	Low risk
**Caumo et al., 2022**	Low risk	Some concerns	Some concerns	Low risk	Some concerns	Some concerns
**Pilotti et al., 2026**	Low risk	Low risk	Low risk	Low risk	Some concerns	Some concerns
**da Cunha et al., 2024**	Low risk	Low risk	Low risk	Low risk	Some concerns	Some concerns
**Low risk, n**	11	14	9	20	3	1
**Some concerns, n**	10	6	8	1	17	14
**High risk, n**	0	1	4	0	1	6

RoB 2 domains: D1, bias arising from the randomization process; D2, bias due to deviations from intended interventions; D3, bias due to missing outcome data; D4, bias in measurement of the outcome; D5, bias in selection of the reported result.

Supplementary Table S2. GRADE Summary of Findings

1. Population: Adults receiving tDCS across eligible clinical and nonclinical populations
2. Intervention: Active transcranial direct current stimulation, alone or with a balanced co-intervention
3. Comparator: Sham transcranial direct current stimulation, alone or with the same co-intervention
4. Follow-up: Immediately after completion of the intervention unless otherwise specified

Outcome	Anticipated effect of active tDCS compared with sham	Studies or comparisons	Risk of bias	Inconsistency	Indirectness	Imprecision	Publication bias	Certainty
**Serum BDNF, post-treatment**	MD −0.01, 95% CI −0.15 to 0.13. Active tDCS may have little or no effect on serum BDNF.	Serum subgroup of 8 BDNF comparisons	Serious[1]	Not serious	Not serious	Serious[2]	Not strongly suspected	Low ⊕⊕◯◯
**Plasma BDNF, post-treatment**	MD 0.02, 95% CI −0.25 to 0.29. Active tDCS may have little or no effect on plasma BDNF.	Plasma subgroup of 4 (259 Participants) BDNF comparisons	Serious[1]	Not serious	Not serious	Serious[2]	Not strongly suspected	Low ⊕⊕◯◯
**Pain intensity, post-treatment**	MD −1.79, 95% CI −2.34 to −1.23. Active tDCS may reduce pain intensity.	3 comparisons	Serious[3]	Serious[4]	Not serious	Not serious	Not strongly suspected	Low ⊕⊕◯◯
**Pain catastrophizing, PCS total score**	MD −5.11, 95% CI −10.33 to 0.12. Active tDCS may reduce catastrophizing, but the evidence includes the possibility of no effect.	3 comparisons	Serious[3]	Not serious	Not serious	Serious[5]	Not strongly suspected	Low ⊕⊕◯◯
**Pain-related disability, PCP:S total score**	MD −11.39, 95% CI −16.67 to −6.12. Active tDCS may reduce pain-related disability.	3 comparisons[6]	Serious[3]	Not serious	Not serious	Serious[7]	Not strongly suspected	Low ⊕⊕◯◯
**Working memory, post-treatment**	No consistent improvement was demonstrated. A quantitative raw MD was not considered clinically interpretable because different instruments and constructs were combined.	7 comparisons	Serious[3]	Not serious[8]	Serious[9]	Serious[10]	Not strongly suspected	Very low ⊕◯◯◯

GRADE footnotes

1. Risk of bias for BDNF: Downgraded one level because most contributing studies had some concerns or high risk of bias, mainly arising from incomplete biomarker outcome data, per-protocol analyses, post-randomization exclusions, exclusion of outcome outliers, crossover carryover concerns, and absence of clearly prespecified BDNF analyses. Contributing reports included substantial attrition in Borzooee et al., post-randomization exclusions in Alcalá-Lozano et al., exclusion of outcome outliers in Adam et al., and incomplete biomarker availability in SELECT-TDCS.
2. BDNF imprecision: Downgraded one level because the confidence intervals included possible increases and decreases in BDNF, the total information size was limited, and no established minimally important difference exists for circulating BDNF.
3. Clinical-outcome risk of bias: Downgraded one level because the evidence was derived predominantly from small trials with concerns regarding allocation concealment, missing outcomes, blinding integrity, selective reporting, or use of estimated summary statistics.
4. Pain-intensity inconsistency: Downgraded one level because considerable and unexplained statistical heterogeneity was present, with $I^2 = 82\%$.
5. PCS imprecision: Downgraded one level because the 95% CI crossed no effect and included both little or no benefit and a potentially important reduction in catastrophizing.
6. Number of PCP:S 102 participants.
7. Disability imprecision: Downgraded one level because the result was based on a small number of trials and did not meet a clearly established optimal information size, despite the confidence interval excluding no effect.
8. Working-memory inconsistency: Statistical heterogeneity was low at $I^2 = 14\%$; therefore, no downgrade was applied for statistical inconsistency.
9. Working-memory indirectness: Downgraded one level because the included measures assessed different aspects of working memory and broader cognitive performance and were not directly interchangeable.
10. Working-memory imprecision: Downgraded one level because the number of participants was limited and the evidence did not exclude either no effect or a potentially meaningful benefit.

Supplementary File S1. The PRISMA checklist.

Section and Topic	Item #	Checklist item	Location where item is reported
**TITLE**			
Title	1	Identify the report as a systematic review.	Page 1
**ABSTRACT**			
Abstract	2	See the PRISMA 2020 for Abstracts checklist.	Pages 1–2
**INTRODUCTION**			
Rationale	3	Describe the rationale for the review in the context of existing knowledge.	Pages 3–5
Objectives	4	Provide an explicit statement of the objective(s) or question(s) the review addresses.	Page 5
**METHODS**			
Eligibility criteria	5	Specify the inclusion and exclusion criteria for the review and how studies were grouped for the syntheses.	Pages 5–8
Information sources	6	Specify all databases, registers, websites, organisations, reference lists and other sources searched or consulted to identify studies. Specify the date when each source was last searched or consulted.	Page 5
Search strategy	7	Present the full search strategies for all databases, registers and websites, including any filters and limits used.	Page 5
Selection process	8	Specify the methods used to decide whether a study met the inclusion criteria of the review, including how many reviewers screened each record and each report retrieved, whether they worked independently, and if applicable, details of automation tools used in the process.	Page 5
Data collection process	9	Specify the methods used to collect data from reports, including how many reviewers collected data from each report, whether they worked independently, any processes for obtaining or confirming data from study investigators, and if applicable, details of automation tools used in the process.	Pages 7-8
Data items	10a	List and define all outcomes for which data were sought. Specify whether all results that were compatible with each outcome domain in each study were sought (e.g. for all measures, time points, analyses), and if not, the methods used to decide which results to collect.	Pages 7-8
	10b	List and define all other variables for which data were sought (e.g. participant and intervention characteristics, funding sources). Describe any assumptions made about any missing or unclear information.	Page 7-8
Study risk of bias assessment	11	Specify the methods used to assess risk of bias in the included studies, including details of the tool(s) used, how many reviewers assessed each study and whether they worked independently, and if applicable, details of automation tools used in the process.	Page 6-7
Effect measures	12	Specify for each outcome the effect measure(s) (e.g. risk ratio, mean difference) used in the synthesis or presentation of results.	Pages 7-8
Synthesis methods	13a	Describe the processes used to decide which studies were eligible for each synthesis (e.g. tabulating the study intervention characteristics and comparing against the planned groups for each synthesis (item #5)).	Pages 7–8
	13b	Describe any methods required to prepare the data for presentation or synthesis, such as handling of missing summary statistics, or data conversions.	Pages 7-8
	13c	Describe any methods used to tabulate or visually display results of individual studies and syntheses.	Page 7-8; Supplementary Figures S1–S9
	13d	Describe any methods used to synthesize results and provide a rationale for the choice(s). If meta-analysis was performed, describe the model(s), method(s) to identify the presence and extent of statistical heterogeneity, and software package(s) used.	Page 8-9

Section and Topic	Item #	Checklist item	Location where item is reported
	13e	Describe any methods used to explore possible causes of heterogeneity among study results (e.g. subgroup analysis, meta-regression).	Page 9
	13f	Describe any sensitivity analyses conducted to assess robustness of the synthesized results.	Page 9
Reporting bias assessment	14	Describe any methods used to assess risk of bias due to missing results in a synthesis (arising from reporting biases).	Page 6-7
Certainty assessment	15	Describe any methods used to assess certainty (or confidence) in the body of evidence for an outcome.	Page 6-7
**RESULTS**			
Study selection	16a	Describe the results of the search and selection process, from the number of records identified in the search to the number of studies included in the review, ideally using a flow diagram.	Pages 9-10
	16b	Cite studies that might appear to meet the inclusion criteria, but which were excluded, and explain why they were excluded.	NA
Study characteristics	17	Cite each included study and present its characteristics.	Pages 11-114
Risk of bias in studies	18	Present assessments of risk of bias for each included study.	Page 15; Figure 2; Supplementary Table 1
Results of individual studies	19	For all outcomes, present, for each study: (a) summary statistics for each group (where appropriate) and (b) an effect estimate and its precision (e.g. confidence/credible interval), ideally using structured tables or plots.	Pages 16–17; Supplementary Figures S1–S9
Results of syntheses	20a	For each synthesis, briefly summarise the characteristics and risk of bias among contributing studies.	Pages 16-17
	20b	Present results of all statistical syntheses conducted. If meta-analysis was done, present for each the summary estimate and its precision (e.g. confidence/credible interval) and measures of statistical heterogeneity. If comparing groups, describe the direction of the effect.	Pages 16-17; Supplementary Figures S1–S9
	20c	Present results of all investigations of possible causes of heterogeneity among study results.	Pages 16-17; Supplementary Figures S1–S9
	20d	Present results of all sensitivity analyses conducted to assess the robustness of the synthesized results.	Pages 16-17; Supplementary Figures
Reporting biases	21	Present assessments of risk of bias due to missing results (arising from reporting biases) for each synthesis assessed.	NA
Certainty of evidence	22	Present assessments of certainty (or confidence) in the body of evidence for each outcome assessed.	Pages 15-16; Supplementary Table 2
**DISCUSSION**			
Discussion	23a	Provide a general interpretation of the results in the context of other evidence.	Pages 18–21
	23b	Discuss any limitations of the evidence included in the review.	Page 21-22
	23c	Discuss any limitations of the review processes used.	Page 21-22
	23d	Discuss implications of the results for practice, policy, and future research.	Page 22

Section and Topic	Item #	Checklist item	Location where item is reported
**OTHER INFORMATION**			
Registration and protocol	24a	Provide registration information for the review, including register name and registration number, or state that the review was not registered.	5
	24b	Indicate where the review protocol can be accessed, or state that a protocol was not prepared.	5
	24c	Describe and explain any amendments to information provided at registration or in the protocol.	NA
Support	25	Describe sources of financial or non-financial support for the review, and the role of the funders or sponsors in the review.	Page 23
Competing interests	26	Declare any competing interests of review authors.	Page 23
Availability of data, code and other materials	27	Report which of the following are publicly available and where they can be found: template data collection forms; data extracted from included studies; data used for all analyses; analytic code; any other materials used in the review.	Page 23; Supplementary files

From: Page MJ, McKenzie JE, Bossuyt PM, Boutron I, Hoffmann TC, Mulrow CD, et al. The PRISMA 2020 statement: an updated guideline for reporting systematic reviews. BMJ 2021;372:n71. doi: 10.1136/bmj.n71. This work is licensed under CC BY 4.0. To view a copy of this license, visit https://creativecommons.org/licenses/by/4.0/
